# Life-History Traits of the Model Organism *Pristionchus pacificus* Recorded Using the Hanging Drop Method: Comparison with *Caenorhabditis elegans*

**DOI:** 10.1371/journal.pone.0134105

**Published:** 2015-08-06

**Authors:** Patricia Gilarte, Bianca Kreuzinger-Janik, Nabil Majdi, Walter Traunspurger

**Affiliations:** Animal Ecology, Bielefeld University, Konsequenz 45, 33615, Bielefeld, Germany; Chinese Academy of Sciences, CHINA

## Abstract

The nematode *Pristionchus pacificus* is of growing interest as a model organism in evolutionary biology. However, despite multiple studies of its genetics, developmental cues, and ecology, the basic life-history traits (LHTs) of *P*. *pacificus* remain unknown. In this study, we used the hanging drop method to follow *P*. *pacificus* at the individual level and thereby quantify its LHTs. This approach allowed direct comparisons with the LHTs of *Caenorhabditis elegans* recently determined using this method. When provided with 5×10^9^ *Escherichia coli* cells ml^–1^ at 20°C, the intrinsic rate of natural increase of *P*. *pacificus* was 1.125 (individually, per day); mean net production was 115 juveniles produced during the life-time of each individual, and each nematode laid an average of 270 eggs (both fertile and unfertile). The mean age of *P*. *pacificus* individuals at first reproduction was 65 h, and the average life span was 22 days. The life cycle of *P*. *pacificus* is therefore slightly longer than that of *C*. *elegans*, with a longer average life span and hatching time and the production of fewer progeny.

## Introduction

Model organisms with short generation times and able to tolerate cultivation under laboratory conditions have long captured the attention of biologists, who have used them to test mechanistic hypotheses at the lowest experimental cost and temporal scale possible [1,2]. For instance, the nematode *Caenorhabditis elegans* Maupas 1900 has for decades served as an animal model for researchers throughout the world [3,4,5]. In the wake of the success of *C*. *elegans*, other Nematoda species have been used to investigate a broader range of traits [6,7].

One example is the nematode *Pristionchus pacificus* Sommer et al. 1996, which holds several important advantages as a model organism such as hermaphroditism, short generation time, and easy cultivation using *Escherichia coli* as a standardized food source [8]. Only in the past five years, 107 publications have focused on *P*. *pacificus* [9], thus revealing its growing use as a model organism.

Providing with an ecological context of model organisms is a crucial point in our understanding of evolutionary biology [10]. *Pristionchus pacificus* has an interesting ecology in its highly host-specific necromenic behavior, a form of phoresy, in which the nematode enters a resting stage once inside its beetle host and awakens upon host death to feed on the ‘soup’ of microbial decomposers growing within the beetle carcass [11,12]. This association is of such intricate nature that horizontal gene transfer from insect to nematode has been reported [13]. Another remarkable ecological trait of *P*. *pacificus* is its ability to switch from a bacterivorous to a predacious phenotype according to physiological and sociological cues [14,15,16]. The unique traits and phenotypic plasticity of *P*. *pacificus* make this nematode a relevant evolutionary model that is likely to yield important molecular, developmental, and ecological insights (e.g. [8,12,14,17,18]).

However, to date, the basic features of *P*. *pacificus* life history, such as its population growth rate and reproductive traits, have not been measured. While genetics determine life-history traits (LHTs) such as fitness and survival, both are inclusively necessary for a broader understanding of animal evolution and population dynamics [19,20]. Thus, for the LHTs of model species, accurate data from empirical measurements are required to establish a robust knowledge of the physiology and evolutionary biology of that species [21]. For instance, the number of offspring and the life span of wild strains recorded under optimal conditions are commonly used as benchmarks to assess divergence in mutant strains or endpoints in ecotoxicological tests [22,23,24].

Multiple nematode culture methods can be used to record LHTs (e.g. [25]). The ‘hanging drop method’ proposed by Muschiol & Traunspurger [26] combines the advantages conferred by culture methods using solid and liquid media in terms of accurately observing, describing, and recording nematode LHTs. This method is based on the use of semi-solid droplets of culture medium that hang from the lid of plastic culture multi-well plates. This set up allows nematodes to be monitored individually over time at any degree of precision. By eliminating intraspecific interferences in batch cultures and the uneven distribution of food items in solid medium, it avoids several sources of error and artefacts.

In this study, we accurately define the major characteristics of the life cycle and LHTs of *P*. *pacificus* strain PS312 under standard conditions. Our methodological approach is based on the ‘hanging drop method’, which is optimal for such purposes. Additionally, we compared the life cycle and LHT of *P*. *pacificus* with those recently obtained from *C*. *elegans* using this same method [27].

## Materials and Methods

### Experimental set-up

*Escherichia coli* (strain OP50) served as the sole food resource for *P*. *pacificus*. Frozen *E*. *coli* were inoculated on LB medium and allowed to grow overnight at 37°C. This inoculum was then used to prepare agar plates for the maintenance of stock cultures on nematode growth medium (NGM). All experimental steps were performed in a sterile environment under a laminar airflow fume hood.

For the experimental set-up, standard *E*. *coli* concentrations were prepared based on the optimal requirements of *C*. *elegans* and *Caenorhabditis briggsae*, which are between 10^9^ and 10^10^ bacterial cells ml^–1^ [28,29]. We therefore used *E*. *coli* concentration of 5×10^9^ cells ml^–1^ as described by Muschiol et al. [27] for *C*. *elegans*, which allowed a direct comparison of its LHTs with those of *P*. *pacificus* determined in this study.

To obtain the desired *E*. *coli* concentration, liquid cultures were centrifuged and the resulting pellets were suspended in K-medium to determine *E*. *coli* density based on the absorbance-to-cell density correlation (OD_600;_ [26]). To facilitate storage, *E*. *coli* suspensions of known concentrations were centrifuged and the pellets were stored in the dark at 8°C for up to 20 days. The pellets were then re-suspended as needed in sterilized semi-liquid nematode growth gelrite medium (NGG), which is analogous to the standard NGM but bacto agar is replaced by a bacterial exopolysacharidic gellam gum (Gelrite, Merck & Co., Kelco Division). This substitution produces a semi-solid medium, enabling nematodes to be individually placed in hanging drops. NGG is similar in appearance and consistency to 0.4% bacto-agar, and can be poured into Petri dishes for the maintenance of cultures (see further details in [26]). Prepared NGG was stored in the dark at 8°C for up to 4 days.

Drops of prepared NGG (8 μl) were placed on the upper lids of sterile 12-well plates. To avoid desiccation of the drops, cellulose gauze pads soaked in distilled water were placed beneath each drop in the corresponding wells. The plates were sealed with Parafilm and incubated in the dark at 20°C. The semi-fluid consistency of the medium allowed the drops to hang on the lids, with evaporation prevented by replacing the wet gauze pads. Individual nematodes could thus be observed uninvasively under a stereomicroscope (×9–90) as required [27].

### Nematode culture maintenance and acclimatization

Cultures of a wild isolate of *P*. *pacificus* (strain PS312) were obtained from the Caenorhabditis Genetics Center (University of Minnesota, Minneapolis, MN, USA). Nematodes were delivered embedded on agar plates containing NGM. Agar plates containing living worms were stored in the dark at 20°C. Approximately every 6 days, the nematodes were transferred to new agar plates previously inoculated with prepared *E*. *coli* suspension. All manipulations of stock cultures were done at 20°C and under sterile conditions. Prior to the experiments, the nematodes were transferred from NGM to NGG and allowed to acclimatize for five days, to reduce potential influences due to acclimatization stress, maternal effects, and contamination.

### Life stage synchronization and experimental run

To obtain accurate LHT measures, nematode life stages were synchronized at inoculation. Ten young adult nematodes with already developed gonads, but non-gravid, were transferred in drops (10 μl) of NGG containing 5×10^9^ *E*. *coli* cells ml^–1^. The nematodes were then left to lay eggs during 30–50 h of incubation in the dark at 20°C. The drops were checked every 3 h. Additional nutritive medium (5 μl) was added daily to provide fresh food and to limit dehydration of the drops. Once the eggs had hatched, juveniles hatched within a 4 h interval (3 h plus 1 h of handling time) were selected for further study. This method ensured that the LHT measures were as temporally accurate as possible.

Synchronized juveniles (n = 33) were gently picked up with a ‘nematode picker’ (one eyebrow hair glued to the tip of a Pasteur pipette) and then individually positioned in their respective drops. Juveniles were checked for life parameters every 24 h, at which time they were transferred to a fresh drop of food medium. When they had reached sexual maturity, egg-laying hermaphrodites were transferred to a fresh drop every 6 h and the number of eggs laid in the drops was recorded. After the adults had been transferred, the drops were left undisturbed for 48 h (originally set to 24 h, which was shown to be insufficient), which allowed the number of hatching vs. non-hatching eggs to be recorded. After 126 h, the maximum fecundity was expected to decline and the worms were transferred to new drops first every 12 h, and then, after 294 h, every 24 h. The experiment was run until the death of the last two remaining adults (after 764 h: ~32 days).

### Data analysis

**Fig 1:**
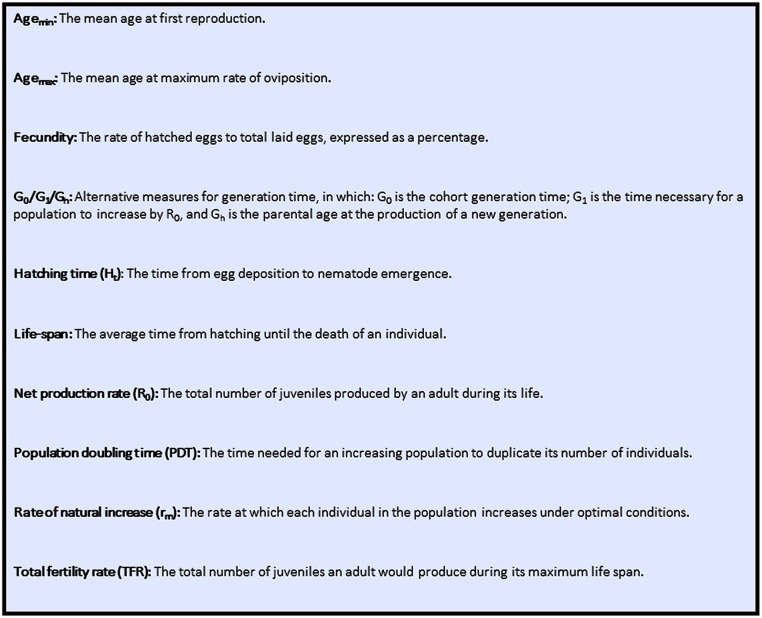
Glossary of terms.

To concisely summarize LHT measures, the data were presented in the form of life tables. The following parameters were calculated: hatching time, net production rate, total fertility rate, life span, generation time, population doubling time, and rate of natural increase. Life span, age at first egg deposition, and maximum rate of egg-laying were expressed as means with their standard deviations. To facilitate interpretation of the results, a glossary of the terms used herein is provided in Fig 1. Hatching time, net production rate, fecundity, rate of natural increase, and alternative measures of generation time were calculated using methods described in the literature and from empirical measurements, as described in detail below:

#### Hatching time

Hatching time was preferred over egg-laying time, as egg-laying can be delayed such that, in extreme cases, intra-uterine hatching may occur (also known as matricidal hatching or *endotokia matricida* [30,31]). Hence, because the experiment started with juveniles and not eggs, a measure of hatching time was needed to accurately assess the entire *P*. *pacificus* life cycle. Our approach was based on the protocol described for *C*. *elegans* by Muschiol et al [27], which was modified to account for the longer developmental period of *P*. *pacificus*.

Briefly, 100 adults and stage-four juveniles of *P*. *pacificus* were randomly picked from an exponential stage culture raised on NGG. The worms were dispatched in ten 20-μl drops of NGG (10 adults per drop) for 32 h, after which the drops were fixed with 4% formaldehyde and stained with Rose Bengal for observation under a stereomicroscope (×90). Hatching time was calculated as:

Ht = Neggs(Njuv+Neggs)×Th

where:

H_t_ = estimated hatching time

N_eggs_ = number of eggs laid during the experimental time

N_juv_ = number of juveniles hatched during the experimental time

T_h_ = experimental time (in hours)

#### Net production rate

The net production rate was calculated as:

$${\text{R}}_{\text{0}}\;=\;\sum_{x\;=\;0}^{d}{\text{l}}_{\text{x}}{\text{ m}}_{\text{x}}$$

where:

R_0_ = net production rate

l_x_ = age-specific survival probability

m_x_ = age-specific fecundity

The value of R_0_ can be interpreted as the average number of offspring produced by an individual of the study population during its entire life [20].

#### Fecundity

The fecundity was calculated individually for all sexually mature, alive individuals and then as an arithmetic mean that was converted into a percentage. This allowed an easier interpretation of the resulting graphs. Note that individual deaths due to natural causes were excluded from the calculation of fecundity as an individual mean; they were, however included in the calculations of survivorship and the rate of natural increase of the population.

#### Intrinsic rate of natural increase

The intrinsic rate of natural increase (r_m_) was proposed by Lotka [32] to measure the growth potential of a population. It is defined as the rate of increase per individual in the absence of adverse conditions [33] and is calculated based on Euler's equation:

$$\sum_{x\;=\;0}^{d}e^{-{\text{r}}_{\text{m}}\text{x}}{\text{ l}}_{\text{x}}{\text{ m}}_{\text{x}}\;=\;1$$

where:

r_m_ = intrinsic rate of natural increase

x = time [d]

l_x_ = age-specific survival probability

m_x_ = age-specific fecundity

This equation, also known as the ‘Lotka equation’ [33], is solved by substitutions of the r_m_ value until the equation equality is met (see [20] for calculation details).

#### Population doubling time

In the nematode LHT literature, the term "population doubling time" (PDT) is very broadly employed but with little explanation. The PDT is the time needed for a growing population to duplicate [34]. As such, in a synchronized population, the PDT is the time needed for the population to double after sexual maturity has been achieved; that is, once reproduction has begun. Hence, in an assessment of the entire life cycle of a population, as was our aim with *P*. *pacificus*, the PDT of a synchronized population requires the prior determination of the mean age at first reproduction. PDT is calculated as:

$$\text{PDT }=\text{ ln}\left(2\right)/{\text{r}}_{\text{m}}$$

where:

PDT = population doubling time

ln = natural logarithm

r_m_ = rate of natural increase

#### Alternative measures of generation time

Generation time is sometimes regarded as a subjective concept due to its dependence on population structure and temporal resolution, hence alternative measurements have been proposed [35]. Following these indications, we measured: G_0_ or cohort generation time; G_1_, which is the time necessary for the increasing population to grow by a factor of R_0_; and G_h_, the mean parental age at which a new generation is produced [35]. To calculate G_0_, G_1_, and G_h_, the following formulas were used:

$${\text{G}}_{\text{0}}\;=\;\sum {\text{xl}}_{\text{x}}{\text{ m}}_{\text{x}}/\sum {\text{l}}_{\text{x}}{\text{ m}}_{\text{x}}$$

where:

x = time [d]

l_x_ = age-specific survival probability

m_x_ = age-specific fecundity

$${\text{G}}_{\text{1}}\;=\;\left({\text{lnR}}_{\text{0}}\right)/{\text{r}}_{\text{m}}$$

where:

R_0_ = net reproductive rate

r_m_ = intrinsic rate of natural increase

$${\text{G}}_{\text{h}}\;=\;\sum {\text{xe}}^{-{\text{r}}_{\text{m}}\text{x}}{\text{ l}}_{\text{x}}{\text{m}}_{\text{x}}$$

where:

r_m_ = intrinsic rate of natural increase

x = time [d]

l_x_ = age-specific survival probability

m_x_ = age-specific fecundity

## Results

The hatching time (H_t_) was 25.3±1.6 h. During the life cycle experiment, 24 h was insufficient for total hatching, as a small number of unhatched eggs showed signs of development. After 48 h, however, all unhatched eggs showed no signs of gastrulation or embryogenesis and were considered as unfertile.

**Fig 2:**
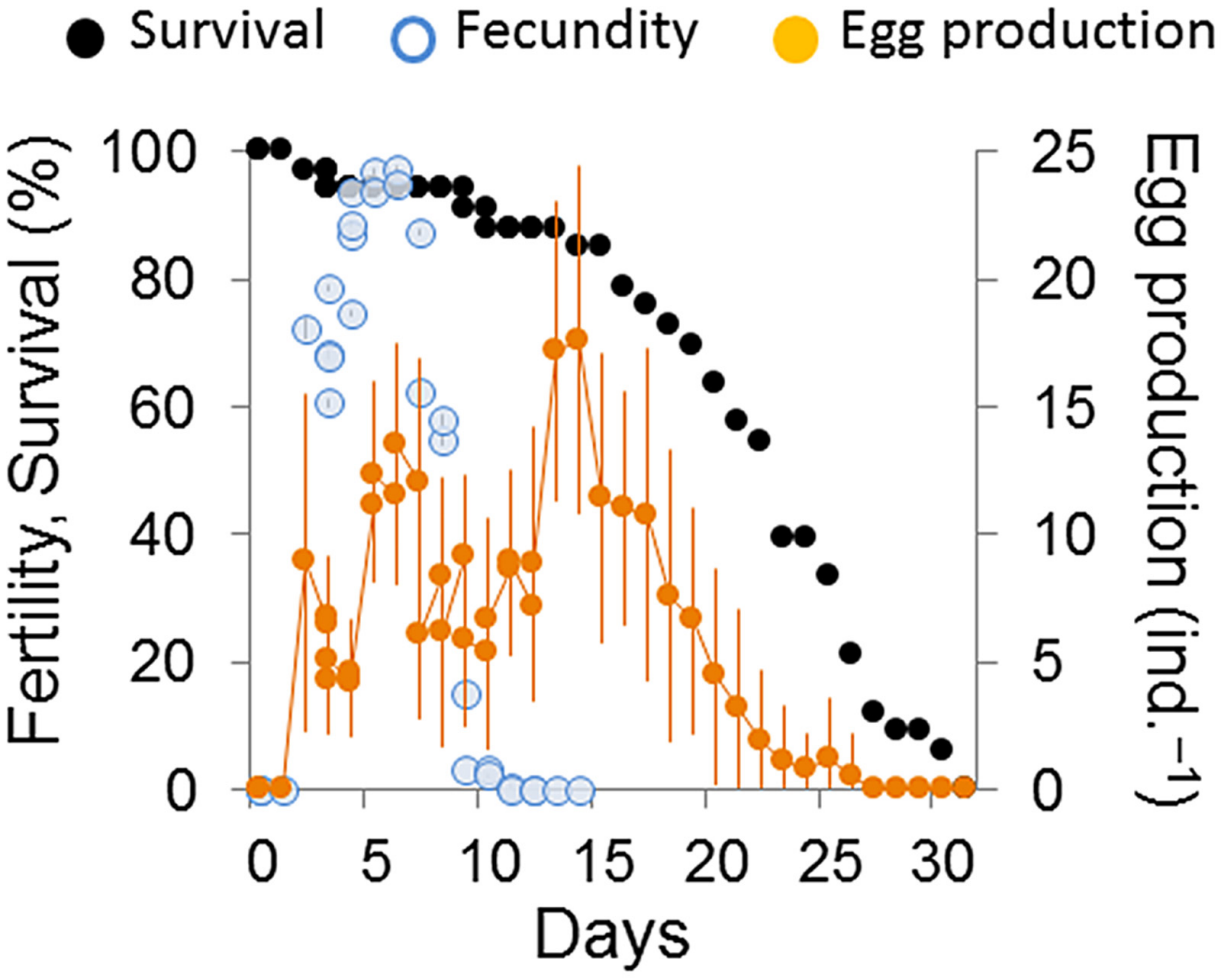
Survival, fecundity and egg production of *Pristionchus pacificus* (strain PS312). Nematodes were cultured at 20°C by means of the hanging drop method. The survival data fit a type I attenuation curve, which is typical in species with a low level of juvenile mortality [36]. Values are reported as the means (N = 33, ±SD).

Most individuals reached sexual maturity; only two died during the juvenile stage (n = 2, 5.6%; Fig 2). These two juveniles became comparatively smaller in the first few days and died after 62 and 89 h. In the remainder of the population, the maximum life span was 32 days, when the last two surviving individuals died. The average length of the population life span was 22.5 ± 7.3 days (Table 1).

**Table 1:** Life cycle parameters of *Pristionchus pacificus* (N = 33).

H_t_ (h)	25.3 ± 1.6
Life span (d)	22.5 ± 7.3
Age_min_ (h)	65.1 ± 8.4
Age_max_(h)	140.8 ± 14.4
TFR	115.35
T_eggs_	269.6 ± 73.4
G_0_	137
G_1_	98
G_h_	80
r_m_	1.125
PDT (h)	14.8
R_0_	108.95

Values are the means ± SD. Abbreviations: H_t_ = hatching time; Age_min_ = age at the start of reproduction; Age_max_ = age at the maximum rate of egg-laying; TFR = total fertility rate (the total number of juveniles produced per nematode during its lifetime); T_eggs_ = total number of eggs produced per nematode during its lifetime; G_0_/G_1_/G_h_ = alternative measures of the generation time; r_m_ = rate of natural increase; PDT = population doubling time; R_0_ = reproductive rate

Reproduction started after an average of 65.1 h (65.1 ± 8.4, Table 1) and lasted until day 13 (Fig 2). During its life span, each nematode laid an average of 270 eggs (269.6 ± 73.4), which resulted in the mean net production of 112 juveniles by all nematodes that reached sexual maturity (112.1 ± 21.1). This corresponded to a mean net production of 109 juveniles per nematode when considering nematode survivorship (R_0_ = 108.95).

The rate of egg-laying peaked twice: between 5 and 7 days, when fecundity was nearly maximum (96%), and at the end of the reproductive period, after 13–14 days, when fecundity had ended (Fig 2). As time resolution was higher during maximum fecundity and early reproduction, the mean number of eggs laid daily was greater in the first peak (25 vs. 18 eggs laid in 24 h, Fig 2).

From the beginning of reproduction, fecundity showed a general positive trend during the first 6 days, corresponding to a maximum fertility rate of 97.4% after 6.1 days. After that, fecundity decreased dramatically such that at day 13 it was negligible, with only one juvenile emerging out of the 257 eggs laid by all living individuals. This corresponded to a fecundity of 0.21% after 12.8 days and null (0%) thereafter.

For *P*. *pacificus*, the TFR (the maximum number of offspring produced by an individual with a maximum life span) was 115 (Table 2). The intrinsic rate of natural increase of the *P*. *pacificus* population was 1.125. The PDT after the start of reproduction averaged 14.8 h (Table 1).

**Table 2:** Life table of *P*. *pacificus* (N = 33).

Time (h)	D (h)	l_x_	m_x_	l_x_m_x_
75	6	0.97	8.34	8.09
81	6	0.97	7.00	6.79
87	6	0.97	5.03	4.88
93	6	0.94	3.61	3.39
99	6	0.94	4.77	4.48
105	6	0.94	3.94	3.70
111	6	0.94	4.63	4.34
117	6	0.94	3.87	3.64
123	6	0.94	4.61	4.33
132	12	0.94	10.81	10.15
144	12	0.94	11.68	10.97
156	12	0.94	11.19	10.52
168	12	0.94	13.16	12.36
180	12	0.94	10.58	9.94
192	12	0.94	4.19	3.94
204	12	0.94	3.81	3.58
216	12	0.94	2.45	2.30
228	12	0.94	1.06	1.00
240	12	0.91	0.30	0.27
252	12	0.91	0.07	0.06
264	12	0.88	0.17	0.15
276	12	0.88	0.03	0.03
288	12	0.88	0.00	0.00
306	24	0.88	0.00	0.00
330	24	0.88	0.03	0.03
		SUM	115.35	108.95

D (h) = time interval; l_x_ = juvenile survival probability; m_x_ = mean number of juveniles produced.

## Discussion

Our results provide the first assessment of the standard life cycle and LHTs of the model nematode *Pristionchus pacificus*. This species is being increasingly used in evolutionary and developmental biology as a ‘satellite’ model organism of *C*. *elegans* [8]. However, until now, an accurate assessment of the standard life parameters of *P*. *pacificus* that allowed a direct comparison with *C*. *elegans* was lacking. The basic measures of standard population metrics reported herein and the use of optimal procedures to culture and observe this nematode provide a robust benchmark for further practical laboratory investigations using *P*. *pacificus* models.

The hanging drop method [26,27] used in this study has several advantages over cultures in solid or liquid medium. First, it allows the accurate calculation and even distribution of bacterial food, because the semi-solid consistency of the NGG drops avoids sinking of the bacteria and worms. Since fresh food is provided periodically to individual worms, optimal conditions and absence of metabolites accumulation and poor oxygenation are assured. The follow up to the individual level allowed the accurate calculation of life parameters. Moreover, the hanging drop method enables observations of individuals through the upper plastic lid of multi-well plates or Petri dishes and thus avoids disturbance of the nematodes. Hence, many additional traits can be recorded (e.g. pumping rate, locomotion), leading to a high-throughput of behavioural data if this method is coupled with automated-image tracking procedures [37,38]. As it can be adjusted to any desired degree of temporal resolution, we were able to obtain accurate data of fertility and egg production at the time of maximum fecundity, when high numbers of eggs and juveniles may interfere during counting. Given its practicability, since first proposed by Muschiol and Traunspurger [27] this method has been successfully used in several studies of nematode LHTs (listed in Table 3).

**Table 3:** LHTs of Rhabditid nematodes recorded using the ‘hanging drop method’.

Species	*Pristionchus pacificus*	*Caenorhabditis elegans*		*Poikilolaimus* sp.	*Panagrolaimus* sp. 1	*Panagrolaimus* sp. 2	*Steinernema riobrave*	
**Food (*E*. *coli* cells ml** ^**–1**^)	5×10^9^	5×10^9^		10^9^		3×10^9^	5×10^9^	
**Temperature (°C)**	20	20		20		21	25	
**Strain/Origin**	**PS312**	**N2**	**MY6**	**Movile Cave (Romania)**		**NFS-24**	**Sr-12**	**Sr-HYB19**
**Individuals monitored**	33	36	36	24	24	24	96	66
**Life span (d)**	22.5	16.7	14.7	-	-	-	7.4	6.9
**Age**_**min**_ **(h)**	65.1	73.0	67.3	468	228	5.7	-	-
**Age**_**max**_ **(h)**	141	108	105	-	-	-	-	-
**G**_**0**_	137	115	106	321	809	247	97	131
**G**_**1**_	98	99	93	324	681	218	142	128
**G**_**h**_	80	90	84	331	629	197	115	126
**r**_**m**_	1.125	1.375	1.460	0.165	0.309	0.530	1.130	1.230
**PDT (h)**	14.8	12.1	11.4	54.0	101.0	31.2	14.9	13.7
**TFR**	115	295	290	187	77	157	975	822
**R**_**0**_	109	291	289	108	64	119	792	683
**Reference**	Present study	[27]		[26]		[39]	[40]	

Values are means. Abbreviations: Age_min_ = age at the start of reproduction; Age_max_ = age at the maximum rate of egg-laying; TFR = total fertility rate; G_0_/G_1_/G_h_ = alternative measures of the generation time; r_m_ = rate of natural increase; PDT = population doubling time; R_0_ = reproductive rate

In agreement with previous findings, the complete reproductive cycle of *P*. *pacificus* was <4 days [41]. However, this estimate includes the hatching time of juveniles. In fact, age at first reproduction was lower in *P*. *pacificus* than in *C*. *elegans*, reported to be a fast reproducer [41]. Nevertheless, the earlier start of *P*. *pacificus* reproduction was compensated for by a much longer hatching time (Table 4). The apparent variation in the literature regarding hatching time can be explained considering constraints, such as food depletion [31], which can delay hatching to the point of matricidal hatching. In old individuals, matricidal hatching may simply reflect malfunction of the egg-laying machinery [42]. This mechanism is not known for *P*. *pacificus* and in this study we observed it only once, in an old hermaphrodite (9.1 days). An egg laid by another old hermaphrodite (9.6 days) hatched less than 12 h after its deposition. Hence, the delayed egg-laying and matricidal hatching observed here in *P*. *pacificus* appear to be consequences of the aging process and thus independent of the food conditions.

**Table 4:** Hatching time (h) for *Caenorhaditis elegans* and *Pristionchus pacificus*.

	*Caenorhaditis elegans*		*Pristionchus pacificus*	Temperature (°C)
Reference/Strain	N2	MY6	PS312	-
[43]	13	-	-	20–22
[44]	18	-	24	20
[27]	7.3	9.9	-	20
Present study	-	-	25	20

Values are means.

The hatching time of 25 h measured in this study agrees with the embryonic development period of approximately 24 h previously reported for *P*. *pacificus* [44]. The relatively longer development time has been attributed to the complexity of the buccal structure of *P*. *pacificus* [45,46]. Additionally, as a member of the Diplogastromorpha, *P*. *pacificus* hatches directly as a second stage juvenile, with the first moult taking place inside the egg [46]. Thus, despite its younger age at first reproduction, *P*. *pacificus* has a slightly longer total life cycle than *C*. *elegans* (3.5 days [47]).

As suggested by Charlesworth, we used alternative measures of the generation time [35]. Surprisingly, there was a large difference between G_0_ (137 h) and G_h_ (80 h) (Table 1). The results of age at first reproduction of 65 hours (65.1 ± 8.4) and PDT of 15 hours (14.8 h) are in concordance with the production of a new generation (G_h_) in 80 hours.

The LHTs of *P*. *pacificus* were compared with those of other rhabditid nematodes assessed using the hanging drop method (Table 3). During its reproductive period, *P*. *pacificus* produced fewer offspring than *C*. *elegans* (109 vs. 290 average offspring per lifespan). As a result, the rate of the natural population increase was lower for *P*. *pacificus* than for *C*. *elegans* (1.125 vs. 1.375; [27]). Nevertheless, the population increases of other rhabditid nematodes, as determined in other studies, were much smaller: For instance, *Panagrolaimus* sp. (strain NFS-24) had a population increase of 0.53 at 21°C [39]. *Panagrolaimus* sp. and *Poikilolaimus* sp., both isolated from a cave ecosystem, had population increases of 0.309 and 0.165, respectively [26]. Only *Steinernema riobrave* (strain Sr 7/12), an entomopathogenic nematode, had a similar population increase of 1.13 at 25°C [40]. Indeed, temperature might affect the dynamics of metabolic processes in poikilothermic small invertebrates such as nematodes. Nevertheless, considering the unique host-specific necromenic ecology of *P*. *pacificus*, which has been proposed as an intermediate step towards parasitism [48], the population increase determined for *P*. *pacificus* is very similar to the values reported for the necromenic *C*. *elegans* and the entomopathogenic nematode *S*. *riobrave*.

Despite its relatively high rate of natural increase, offspring production by *P*. *pacificus* was similar to that of slower reproducers such as *Panagrolaimus* spp. [26,39]. A possible explanation is the relatively large number of unfertile eggs produced by *P*. *pacificus* hermaphrodites (270 eggs over a life-time, 56 of which were laid after the overall fecundity was null). Since *P*. *pacificus* is a self-fertilizing hermaphrodite with a limited amount of sperm, a larger number of progeny would be expected in the presence of males [49], such as occurs in wild populations or batch cultures.

Juvenile mortality in *P*. *pacificus* was minor; the few juveniles that did not reach sexual maturity were comparatively smaller than those able to mature. Observations of the second generation of juveniles were beyond the scope of this study; however, we did note that old adults (>8 days) with a low fecundity produced smaller offspring and in some cases seemingly aberrant nematodes.

The food source in this study was OP50 *E*. *coli*, as in laboratory protocols in which *P*. *pacificus* served as the model organism [50]. In the wild, *P*. *pacificus* shows an omnivorous, necromenic life style [8]. This facultative switch to a scarab beetle host brings complexity into the life cycle of this nematode, which can behave both as a bacterial feeder and as an antagonistic entomophile [51]. Since entomopathogenic nematodes are highly dependent on temperature [52] and achieve higher progeny and growth rates with increasing food concentrations [40], reproductive values for *P*. *pacificus* higher than those determined in this study may be possible at higher food concentrations and temperature. Further studies are needed to shed more light into the complex ecology of this model organism.

## Conclusions

This was the first study to measure the LHTs of the model organism *P*. *pacificus* based on observations during culture of this nematode using the hanging drop method. Compared to *C*. *elegans*, *P*. *pacificus* has a slightly longer life cycle, a longer life span, and longer hatching times and produces fewer progeny under standard conditions of food and temperature (5×10^9^ *E*. *coli* cells ml^–1^; 20°C). Although the singular ecology of *P*. *pacificus* prevents the extrapolation of our results to the fitness and performance of this species *in natura*, we believe that our LHT measures provide a solid reference in further investigations using this model species.
